# First-Line Combination with Proteasome Inhibitor-Based Treatment and Zoledronic Acid Is Effective in Reducing Later Fractures in Multiple Myeloma Irrespective of Multiple Myeloma Bone Disease at Diagnosis

**DOI:** 10.3390/hematolrep16030051

**Published:** 2024-08-06

**Authors:** Veera Eskelinen, Elise Nivakoski, Kirsi Launonen, Anu Partanen, Sakari Kakko, Milla E. L. Kuusisto

**Affiliations:** 1 Department of Medicine, University of Oulu, 90220 Oulu, Finland; veera.eskelinen@student.oulu.fi (V.E.); elise.nivakoski@fimnet.fi (E.N.); sakari.kakko@pohde.fi (S.K.); 2 Department of Hematology, Oulu University Hospital, 90220 Oulu, Finland; kirsi.launonen@pohde.fi; 3 Department of Hematology, Kuopio University Hospital, 70290 Kuopio, Finland; anu.partanen@pshyvinvointialue.fi; 4 Cancer and Translational Research Unit, University of Oulu, 90220 Oulu, Finland; 5 Department of Internal Medicine, Länsi-Pohja Central Hospital, 94100 Kemi, Finland; 6 Medical Research Unit, Oulu University Hospital, 90220 Oulu, Finland

**Keywords:** multiple myeloma, skeletal-related events, proteosome inhibitor, zoledronic acid, bone disease

## Abstract

The present study provides real-world evidence on the treatment of multiple myeloma (MM) bone disease with various bisphosphonates combined for different myeloma-specific treatments as no validated data regarding the best combination treatment for bone disease associated with MM are available. We examined retrospectively 345 MM patients treated with autologous stem cell transplantation in Finland during 1996–2020. The median age of the patients was 60 years with a median follow-up time of 50 months (1–339). At diagnosis, 72.1% of the patients had myeloma-associated bone disease and 45.8% had fractures. Most patients (58.8%) received proteasome inhibitor (PI)-containing treatment at first line. MM bone disease was treated in 91.6% of the patients; 49.9% received zoledronic acid (ZA) and 29.9% pamidronate. Inferior overall survival was associated with MM bone disease at diagnosis (*p* = 0.005) or a fracture at diagnosis (*p* = 0.003). A later fracture was identified in 29% of the patients, and in those patients without MM bone disease at diagnosis later fractures were less common after ZA treatment (*p* = 0.049). PI-based treatment plus ZA (*p* = 0.019) seemed to be the best combination to prevent later fractures, even though the same patient subgroup was more likely to experience relapse (*p* = 0.018), and also when excluding patients with previous induction therapy without novel agents (*p* = 0.008). To conclude, this study suggests that the best therapy to prevent later fractures in MM might be PI-based treatment combined with ZA.

## 1. Introduction

Multiple myeloma (MM) is an incurable plasma cell disorder that accounts for up to 10% of all hematological neoplasms and 1–1.8% of all cancer cases [1]. MM to be treated is characterized currently by CRAB criteria (hypercalcemia, renal failure, anemia and bone lesions) and/or specific biomarkers, including bone marrow plasma cells of 60% or greater, a serum free light chain ratio of 100 or greater and more than one focal lesion at least 5 mm in size on MRI studies [2]. The life expectancy after MM diagnosis varies from a few months to over 10 years [3]. Since MM is inevitably a fatal disease, the development of bone disease treatment could significantly improve patients’ quality of life as well as life expectancy.

At the time of diagnosis, most patients are diagnosed with bone abnormalities such as osteolytic lesions, osteopenia/osteoporosis and bone fractures [4,5]. MM-related bone disease is one of the key characteristics of MM. Increased osteoclast activity and reduced osteoblast function cause osteopenia/osteoporosis and osteolytic lesions, evidently leading to bone fractures. At diagnosis, osteolytic lesions are detected in 70 to 80% of patients [2,5]. The occurrence of plasmacytomas is relatively common with MM patients. The appearance of plasmacytomas, including both bone plasmacytoma and extramedullary plasmacytoma, varies from 0.5 to 4.8% with newly diagnosed MM and from 3.4 to 14% with relapsed disease [6]. Radiation therapy is used to treat patients with solitary plasmacytoma but without systemic disease or to treat patients with symptomatic plasmacytoma. Plasmacytomas may lead to pathological fractures or spinal cord compression, which alongside radiation therapy may need to be treated with surgical interventions [6].

By tradition, bisphosphonates such as zoledronic acid (ZA) and pamidronate have been the number one choice for MM-related bone disease treatment. The International Myeloma Working Group (IMWG) recommends considering denosumab alongside bisphosphonates in the treatment of MM-related bone disease due to an improved understanding of the mechanism behind the bone disease and up-to-date research data. Bisphosphonates relieve pain and prevent skeletal-related events (SRE) and spinal cord compression [7]. Vitamin D deficiency is characteristic for MM patients, and, to prevent hypocalcaemia, vitamin D and calcium supplements should be administered to all patients treated with bisphosphonates [8]. In 2010, Mhaskar et al. suggested that ZA is more effective than pamidronate in the prevention of skeletal events [9]. ZA is currently used more than pamidronate [10], and the International Myeloma Study Group recommends using ZA over pamidronate [8]. Still, the existing research data provide limited information comparing the efficacy of ZA and pamidronate in the treatment of MM-related bone disease.

In this study, we aimed to retrospectively determine the best combination of anti-myeloma and bone disease treatment to prevent SRE on patients. In addition, we wanted to compare the effectiveness of two bisphosphonates, ZA and pamidronate, in the treatment of MM-related bone disease. The current research data offer up-to-date evidence of neither the impact of different bisphosphonates nor the treatment combination of anti-myeloma drugs and bisphosphonates in the prevention of SREs.

## 2. Materials and Methods

The patient material was collected from the autological stem cell transplant (ASCT) registry consisting of patients treated in the Oulu University Hospital during 1996–2020. A total of 345 patients were included in the analysis (Table 1). The inclusion criteria for the study included transplant-eligible patients, with an ECOG status of 2 or more acting as the exclusion criterion for the transplant, and these patients were excluded from the study. From the medical records, we retrieved data on diagnosis status, comorbidities, MM follow-up tests, SREs, and the occurrence of adverse reactions in used MM-related bone disease treatments and on treatment efficacy. There were 12.7% of patients (n = 44) who had two comorbidities and one had three comorbidities. Patients’ risk categories were defined by using ISS, R-ISS and IMWG, but unfortunately limited data in the standard workup caused a limitation to stage the patients into risk categories in 41.2%, 63.8% and 60.3% of patients, respectively. The cytogenetics of the myeloma were evaluated from patients by FISH from the bone marrow samples. Patients were representative of the normal MM group as all risk categories were represented, and their prevalence was similar to that reported in the literature. MRIs, CTs or X-rays were used to diagnose the MM-related bone disease. Later fracture was defined as a fracture that appeared no earlier than three months after MM diagnosis or during progressive disease. Later fracture was diagnosed by MRI, CT or X-ray. Bone density measurement results were evaluated as normal, osteopenia or osteoporosis. Normal bone density was defined to be from +1 to −1 SD, osteopenia from −1 to −2.5 SD and osteoporosis at −2.5 SD or lower. In this study, the principles of the Declaration of Helsinki were applied. Ethical decisions were made following the regulation of the Local Ethics Committee of the Northern Ostrobothnia Hospital District.

Patient data were collected in IBM SPSS Statistics 27.0 for Windows. Both IBM SPSS Statistics 27.0 for Windows and Rstudio 2022.02.3 were used for data analysis. Overall survival (OS) and progression-free survival (PFS) were calculated as the time from diagnosis to last follow-up date or progression or death from any cause, whichever came first. Time to next fracture (TTNF) was calculated from diagnosis to last follow-up date or next fracture, whichever came first. The follow-up time was calculated as months from the date of diagnosis to last follow-up date.

Nominal variables were calculated with a chi-square and Fisher’s exact test. Uni- and multivariate analysis was carried out with ANOVA and Cox regression tests. Survival was calculated with a long-rank test. Values of *p* < 0.05 were considered statistically significant.

## 3. Results

Of the patients included in this study, 54.8% were male (n = 189) and 45.2% were female (n = 156) (Table 1). Due to a missing exact diagnosis date, four patients were excluded from this count. The follow-up time ranged from 1 to 339 months with a median of 50 months. The cytogenetics were available from 47.1% of the patients; 33.3% presented standard risk mutations, 7.2% a possible high risk mutation (dup1) and 6.6% high risk mutations such as dep17p, t(4;14) and t(4;18). Patients’ treatment information is summarized in Table 2.

**Figure 1:**
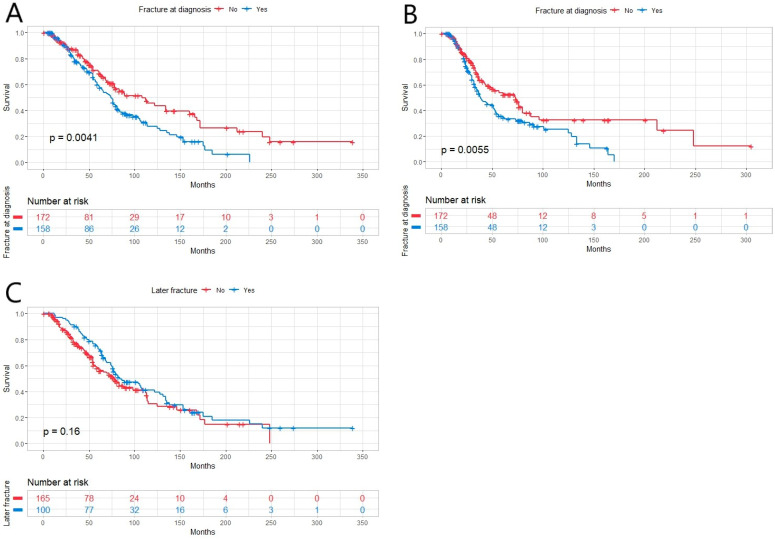
(**A**) Overall survival (OS) in newly diagnosed multiple myeloma patients (n = 345) with fracture at diagnosis (n = 158) was inferior compared to ones without fracture (n = 172, *p* = 0.0041). (**B**) Progression-free survival (PFS) was inferior in patients with fracture at diagnosis (*p* = 0.0055). (**C**) Later fracture had no effect on OS (*p* = 0.16).

At time of diagnosis, 72.8% (n = 251) of the patients were diagnosed with MM-related bone disease and 45.8% (n = 158) had one or multiple fractures, from which 29.3% were pathological (n = 101) and 15.7% osteoporotic (n = 54). One hundred patients (29%) were diagnosed to have a later fracture with a median time to later fracture of 38 months (range 1–187 months). With the fracture on vertebra or ribs, the median for OS was 81 months, whereas with fractures on other locations, the median was 59 months (*p* = 0.015). The quality of fracture, whether pathological or osteoporotic, had no statistically significant effect on OS (*p* = 0.380). Whether the fracture at diagnosis was osteoporotic or pathological, it had no statistically significant impact on the median time to new fracture (*p* = 0.988). Overall, the median time to new pathological fracture and to new osteoporotic fracture had no statistically significant difference (*p* = 0.881). The quality of fracture, osteoporotic or pathologic, was defined by a radiology specialist. Fractures at diagnosis impaired OS and PFS (Figure 1A,B) but later fractures had no impact on OS (Figure 1C).

**Figure 2:**
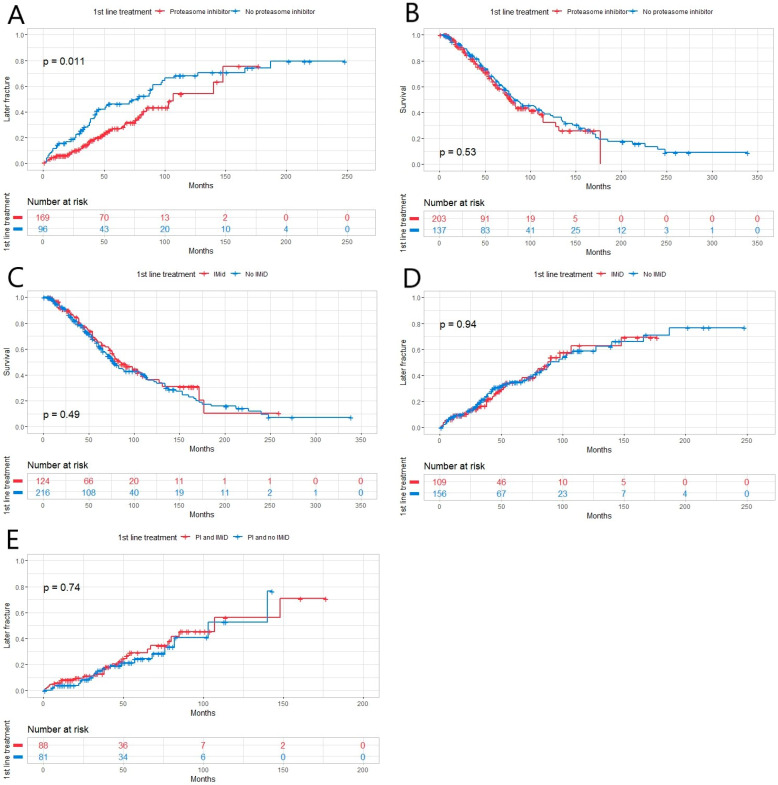
(**A**) Patients with multiple myeloma who received treatment containing proteasome inhibitor (PI) in the 1st line (n = 203) had fewer later fractures than those who did not receive PI (n = 137, *p* = 0.011). (**B**,**C**) There were no statistically significant differences in patients’ OS between different 1st-line treatment groups with/without PI (with n = 203) or an immunomodulatory drug (IMiD) (with n = 103, *p* = 0.53, *p* = 0.49, respectively). (**D**) There was no statistically significant difference between the two treatment groups (with or without IMiD) when comparing the incidence of later fracture (*p* = 0.94). (**E**) There was no statistically significant difference (*p* = 0.74) between patients treated with PI and IMiD (n = 101) compared to those treated with PI and without IMiD in the incidence of later fractures (n = 102).

In the 1st line, 58.8% of the patients (n = 203) were treated with PI, 30.4% with IMiD (n = 105) and 29.3% (n = 101) received the treatment combination of the two mentioned above. First-line bone disease treatment was administered for 91.6% (n = 316) of the patients. The most-used bisphosphonates in the first line were ZA with 49.9% (n = 172) and pamidronate with 29.9% (n = 103) of patients. Treatment with PI in the first line showed a positive effect on inhibiting later fractures (Figure 2A) but not on OS (Figure 2B). In multivariate analysis, there were no independent risk factors to predict later fractures. When first-line treatment with and without IMiD was compared, no statistically significant impact on OS or later fractures was found (Figure 2C,D). Treatment containing PI and IMiD showed no superiority to treatment with PI and without IMiD in the prevention of later fractures (Figure 2E). Calcium with vitamin D substitution in the first line was administered for 63.8% (n = 220).

**Figure 3:**
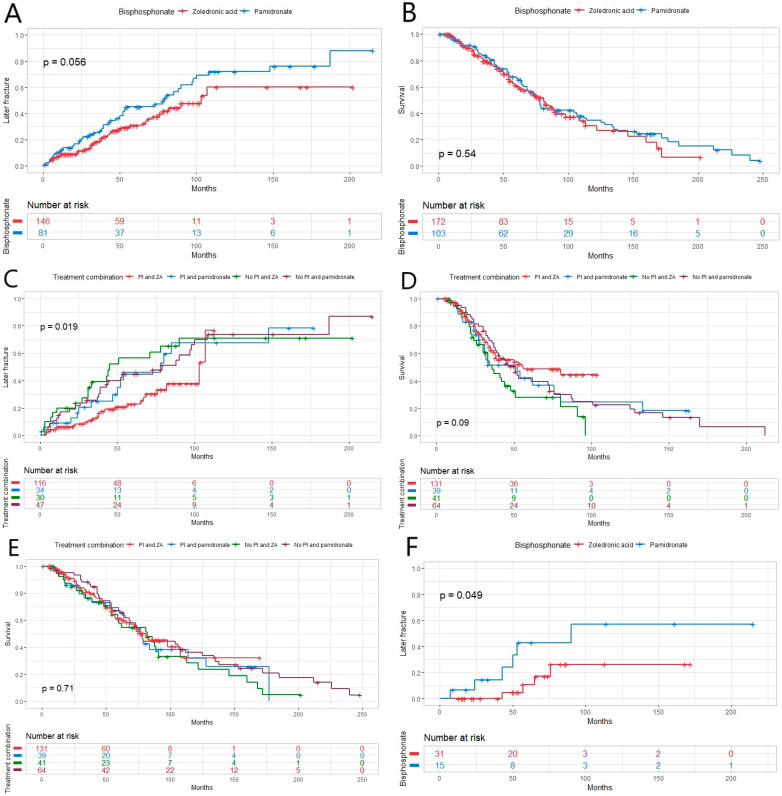
(**A**) There was almost a statistically significant difference between the two bisphosphonates zoledronic acid (ZA) and pamidronate and their effect on later fracture in this newly diagnosed multiple myeloma (MM) patient population (*p* = 0.056), and (**B**) in life expectancy between the bisphosphonates (*p* = 0.54). (**C**) The time to next fracture was longer in patients receiving proteasome inhibitor (PI)-containing treatment combined with ZA (*p* = 0.019). (**D**) There was no effect on progression-free (*p* = 0.09) or (**E**) overall survival between different treatment combinations in the same patient group (*p* = 0.71). (**F**) Within patients who did not have MM-related bone disease at diagnosis, there was a statistically significant difference between two bisphosphonates and their effect on later fracture (*p* = 0.049).

When comparing the effect of two bisphosphonates, ZA and pamidronate, on preventing later fractures, the superiority of ZA did not quite reach a statistically significant result (Figure 3A). However, when comparing the treatment combination of PI and bisphosphonates for preventing later fractures, the combination of PI and ZA appeared to be the best at preventing later fractures (Figure 3B). A difference was found between ZA and pamidronate when comparing their effects on the occurrence of later fractures in patients who did not have bone disease at diagnosis: patients treated with ZA had a longer time to later fracture (Figure 3F). The same patient group treated with PI and ZA were more likely to have a relapse in univariate analysis (*p* = 0.034) as well as in multivariate analysis (*p* = 0.018), even when excluding patients receiving VAD induction from the analysis (*p* = 0.045 and 0.008), respectively (Table 3). There was no statistically significant difference in OS between the two bisphosphonates (Figure 3C). There was no difference in OS or PFS when comparing different combination treatments either (Figure 3D,E).

A total of 63 patients (18.3%) had one or more of the following osteoporosis-predisposing conditions: hypogonadism, rheumatoid arthritis, primary hyperparathyroidism, diabetes mellitus, chronic renal insufficiency, hyperthyroidism, Cushing’s disease, chronic liver disease, celiac disease, inflammatory bowel disease, a postoperative stage of stomach removal, severe lactose intolerance or organ transplant. Treatment for the diagnosed MM-related bone disease was administered every 4 weeks during the first year and every 3 months during the second year after diagnosis. Denosumab was given once a month. The treatment was successfully carried out within patients in this study. Vitamin D content was measured from only 20 patients at diagnosis and the mean for these measurements was 85.1 nmol/L (75–250 nmol/L). Bone density measurements were not performed routinely on all patients. In 45 patients (13.0%), the bone density measurement was performed one or more times. Normal bone density was diagnosed in 11 patients (3.2%), osteopenia in 21 patients (6.1%) and osteoporosis in 13 patients (3.8%). No further analysis of these results can be made because of the small number of patients with bone density measurement results.

## 4. Discussion

In the present study, we found out that the most favorable treatment option for preventing later fractures would be a proteasome inhibitor-based treatment combined with zoledronic acid. PIs have proved their efficiency over the past 15 years in many studies [11], and in the treatment and prevention of MM-related bone disease, bisphosphonates, and of those especially ZA, are the cornerstone [7]. However, limited data were available supporting the combination of these two mentioned above.

In the Cochrane review, 24 studies compared different bisphosphonates to placebo or to no treatment [12]. Our results are consistent with these as bisphosphonates did not predict better OS. Only when ZA was compared to etidronate and placebo alone it appeared to improve OS. However, the incidence of fractures was reduced with the use of bisphosphonates. In contrast to the current study, in the previously mentioned study, from patients receiving ZA, only 11% were treated with ASCT, and from patients receiving pamidronate, 15.6% were treated with ASCT. A cohort study published in 2015 was one of the first in which ZA was directly compared to pamidronate [13]. It was found that ZA was associated with increased OS and fewer fractures when compared to pamidronate. Another study compared ZA to clodronic acid, and patients receiving ZA improved their PFS but not OS [14,15]. In our study, the patients receiving PI and ZA had better outcomes with fewer later fractures than the patients given PI and pamidronate, even though PI and ZA-receiving patients were more likely to have relapsed disease. Cytogenetics showed elevated risk in univariate analysis but not in multivariate analysis, probably because the risk status was only available under half of the studied population. In addition, within patients who were not diagnosed with MM-related bone disease, ZA showed superiority to pamidronate in the prevention of later fractures. This result supports the Swedish National Guidelines from 2010, which recommended all MM patients be treated with bisphosphonates irrespective of the presence of MM-related bone disease [4]. In a retrospective cohort study in 2015, ZA appeared to reduce the risk of death by 22% compared to pamidronate [9]. However, the current study did not present the superiority of ZA in terms of OS. The IMWG recommends bisphosphonates to be initiated for all patients with MM receiving anti-myeloma therapy regardless of the detection of osteolytic bone lesions on conventional radiography as well as for patients with osteoporosis or osteopenia [8]. They also recommend using ZA over pamidronate. The American Society of Clinical Oncology suggests intravenous bisphosphonate use in the case of bone disease in myeloma [16]. However, the data on the impact of the combination of various myeloma-specific treatments with bisphosphonates on patient outcomes are scarce, and neither of the recommendations take a stand on that.

Not many studies have been conducted in order to examine the role of anti-myeloma treatment combined with bone disease treatment to prevent SREs. The phase IB study was published in 2014, which examined the monoclonal antibody BHQ880, with a combination of anti-myeloma treatment and ZA. BHQ880 has showed activity both in anti-myeloma as well as in bone density improvement [17]. The results of the phase II trial have not yet been published. A large multicenter trial was conducted in order to study the effect of denosumab compared to ZA in MM patients to prevent SREs. Denosumab was non-inferior to ZA, but anti-myeloma treatment schemas were decided by the individual investigator’s choice [18]. In the present study, only 3.5% of the patients received denosumab, which precludes further examination.

SREs decline patients’ quality of life (QoL) remarkably. Not many trials have yet assessed QoL aspects (e.g., pain) caused by SREs combined with treatment aggressiveness, but a study was published in 2018 in which the more aggressive the therapy was, the better were the QoL results in terms of pain symptoms caused by SREs [19]. This study was conducted at the same time as VAD induction therapy, but the other arm received ASCT, and all of the patients had ZA as a bone-disease treatment [19]. Even though QoL was not reported in the current study, this would be an important goal for further research to study the impact of bone-disease treatment on the patient’s QoL.

The main limitation of our study is that it is a retrospective study. The data collection was made over a long period of time during which the treatment practices and guidelines have changed and evolved, and a great amount of clinical data were not available. Sociodemographic factors (exc. age) were not collected during the data collection. There were data limitations for the risk assessment evaluation of ISS, R-ISS and IMWG status. The bone disease diagnostics used in almost half of the study population were not those which are currently recommended and therefore it is probable that not all MM-related bone diseases were detected. Patients were not treated following only one or a few different treatment combinations but several different anti-myeloma and PI combinations. There were 36% of patients who received VAD induction, which has no relevance in today’s management of myeloma. Only 8% of patients received an induction regimen that included lenalidomide since lenalidomide does not have reimbursement in the first line in Finland. Also, further analysis concerning bone density could not be made because of the small number of patients with bone density measurement results.

The strengths of the study included the relatively comprehensive patient material and how all transplant-eligible patients received ASCT, and all of them represented the same age group. From the database, we were able to collect extensive clinical data concerning patients’ diagnostic statuses and their treatments. Long follow-up times ensured that comprehensive clinical data were available for patients and improved the reliability of tables and results.

The present study highlights the effectiveness of PI-based therapy combined with bisphosphonates in order to prevent new bone lesions and, most importantly, to prolong overall survival. PI bortezomib seemed to be an important part of the novel induction therapies in transplant-eligible myeloma patients. Larger prospective series of studies are warranted to validate this finding. Altogether, the prevention of SREs in MM can be improved.

## 5. Conclusions

To conclude, the best therapy to prevent later fractures in MM seemed to be PI-based treatment combined with ZA for the benefit of overall survival. To validate the results, a prospective study on the most favorable anti-myeloma and bisphosphonate treatment combination should be performed.

## Figures and Tables

**Table 1:** Patient demographics.

Characteristics	Patients (n = 345)	%
Age at diagnosis		
Median (years)	61	
Range (years)	25–74	
Sex		
Male	189	54.8%
Comorbidities		
Cardiovascular disease	127	36.8%
Diabetes	40	11.6%
Rheumatoid arthritis	10	2.9%
Previous treated cancer	16	4.6%
IBD	8	2.3%
Stroke or TIA	5	1.5%
COPD	4	1.2%
Neurological disease	4	1.2%
Bone disease at diagnosis		
Yes	251	72.8%
No	90	26.1%
Unknown	4	1.1%
Method used to diagnose bone disease		
X-ray	110	43.8%
CT	84	33.5%
MRI	49	19.5%
Unknown	8	3.2%
Fracture at diagnosis		
Yes	158	45.8%
No	172	49.9%
Unknown	15	4.3%
Type of fracture		
Pathological	101	29.3%
Osteoporotic	54	15.7%
Unknown	190	55.1%
Site of fracture		
Vertebra and ribs	122	77.2%
Other	36	22.8%
1st line bone disease treatment		
Yes	316	91.6%
No	15	4.3%
Unknown	14	4.1%
1st line bone-targeted treatment		
No treatment	35	10.1%
ZA	172	49.9%
Pamidronate	103	29.9%
Alendronate	6	1.7%
Denosumab	12	3.5%
Calcitonin	1	0.3%
Unknown	16	4.6%
1st line calcium + vitamin D substitution		
Yes	220	63.8%
No	109	31.6%
Unknown	16	4.6%
Denosumab at 1st line		
Yes	12	3.5%
No	317	91.9%
Unknown	16	4.6%
Later fracture during FU		
Yes	100	29.0%
No	165	47.8%
Unknown	80	23.2%

COPD—chronic obstructive pulmonary disease; FU—follow-up; IBD—inflammatory bowel disease; TIA—transient ischemic attack; ZA—zoledronic acid.

**Table 2:** Patients’ antimyeloma treatment information.

Treatment	Patients (n = 345)	%
1st line PI		
Yes	203	58.9%
No	137	39.7%
Unknown	5	1.4%
1st line IMiD without PI		
Yes	105	30.4%
No	240	69.6%
1st line PI and IMiD		
Yes	101	29.3%
No	239	69.3%
Unknown	5	1.4%
1st line induction PI		
VD	80	23.2%
VCD	74	21.4%
VRD	20	5.8%
VTD	1	0.3%
IRD	7	2.0%
1st line induction IMiD without PI		
Tal-Dex	27	7.8%
Len-Dex	2	0.6%
Other induction therapy		
VAD	123	35.7%
MP	5	1.4%
Cyclo-Dex	1	0.3%
Unknown	5	1.4%
1st line maintenance		
α-interferon	37	10.7%
Tal	6	1.7%
Len	54	15.7%
Single Vel	3	0.9%
Cyclic Dex	1	0.3%
No maintenance	186	53.9%
Unknown	54	15.7%

Cyclo-Dex—cyclophosphamide-dexamethasone; IMiD—immunomodulating drug; IRD—ixazomib-lenalidomide-dexamethasone; Len-Dex—lenalidomide-dexamethasone; MP—melphalan-prednisolone; PI—proteosome inhibitor; Single Vel—bortezomib; Tal-Dex—thalidomide-dexamethasone; VAD—vincristine-doxorubicin-dexamethasone; VCD—bortezomib-cyclophosphamide-dexamethasone; VD—bortezomib-dexamethasone; VRD—bortezomib-lenalidomide-dexamethasone; VTD—bortezomib-thalidomide-dexamethasone.

**Table 3:** Uni- and multivariate analysis comparing proteasome inhibitor and zoledronic acid combination as regards later A. skeletal-related events and B. relapse. Test variables were selected according to relevant induction treatment agents.

	HR	95% CI	*p* Value	*p*-Value ¨
A.				
Cytogenetics	1.112	0.641–1.928	0.706	0.311
PI	1.679	1.121–2.517	0.012	<0.001
PI and IMiD	1.255	1.025–1.536	0.027	<0.001
IMiD	1.016	0.678–1.523	0.937	0.937
PI and ZA	1.252	1.061–1.476	0.008	<0.001
Bisphosphonate	1.503	0.985–2.294	0.060	<0.001
*				
Cytogenetics	1.339	0.758–2.365	0.315	0.634
PI	1.371	0.185–10.137	0.757	0.336
PI and IMiD	1.138	0.663–1.956	0.639	0.271
IMiD	1.108	0.548–2.239	0.775	0.611
PI and ZA	1.451	0.851–2.475	0.171	0.084
Bisphosphonate	2.245	0.953–5.285	0.064	0.114
B.				
Cytogenetics	1.360	1.018–1.816	0.037	0.473
PI	1.009	0.773–1.317	0.949	<0.001
PI and IMiD	1.026	0.899–1.170	0.708	<0.001
IMiD	1.320	0.999–1.744	0.051	0.018
PI and ZA	1.451	1.065–1.976	0.034	0.018
Bisphosphonate	0.941	0.677–1.232	0.553	<0.001
*				
Cytogenetics	1.394	1.035–1.878	0.029	0.760
PI	1.375	0.337–5.615	0.657	0.198
PI and IMiD	1.349	0.986–1.845	0.062	0.043
IMiD	1.459	0.972–2.189	0.068	0.198
PI and ZA	1.516	1.117–2.058	0.045	0.008
Bisphosphonate	1.594	0.906–2.805	0.106	0.303

HR—hazard ratio; IMiD—immunomodulatory drug; PI—proteasome inhibitor; ZA—zoledronic acid; 95% CI confidence interval; * VAD induction patients not included; ¨ multivariate analysis.

## Data Availability

The original data are available by reasonable request.
